# Discovery of Therapeutic Candidates for Diabetic Retinopathy Based on Molecular Switch Analysis: Application of a Systematic Process

**DOI:** 10.1155/2022/3412032

**Published:** 2022-01-06

**Authors:** Yue Ren, Yanan Liu, Kaiyang Liu, Xiaoqian Huo, Chaoqun Liu, Yanling Zhang

**Affiliations:** Key Laboratory of TCM-Information Engineer of State Administration of TCM, School of Chinese Materia Medica, Beijing University of Chinese Medicine, Beijing, China 102488

## Abstract

The pathogenesis of diabetic retinopathy (DR) is complicated, and there is no effective drug. Oxidative stress-induced human retinal microvascular endothelial cells (HRMECs) injury is one of the pathogenic factors for DR. Molecular switches are considered high-risk targets in disease progression. Identification of molecular switch is crucial to interpret the pathogenesis of disease and screen effective ingredients. In this study, a systematic process was executed to discover therapeutic candidates for DR based on HRMECs injury. First of all, the molecular mechanism of HRMECs oxidative stress injury was revealed by transcriptomics and network pharmacology. We found that oxidative stress was one of the pivotal pathogenic factors, which interfered with vascular system development, inflammation, cell adhesion, and cytoskeleton damaged HRMECs through crosstalk. Then, network topology analysis was used to recognize molecular switches. The results indicated that the Keap1-Nrf2-ARE signaling pathway was the molecular switch in HRMECs oxidative stress injury. On this basis, the HEK293-ARE overexpression cell line was applied to obtain 18 active traditional Chinese medicine (TCM) ingredients. Furthermore, andrographolide, one of the 18 candidates, was applied in the HRMECs oxidative stress model to evaluate the accuracy of the systematic process. The efficacy evaluation results showed that andrographolide could regulate oxidative stress, vascular system development, inflammation, adhesion, and skeleton tissue to inhibit HRMECs injury cooperatively. And its mechanism was related to the Nrf2 signaling pathway. Overall, our data suggest that the Nrf2 signaling pathway is the molecular switch in the HRMECs oxidative stress injury. 18 potential Nrf2 agonists are likely to be promising DR candidates.

## 1. Introduction

Diabetic retinopathy (DR) is a common microvascular complication of diabetes. Epidemiological studies have shown that the global prevalence of DR was 27% from 2015 to 2019 [1]. With the increase of DR patients, the visual impairment caused by DR has become severe [2]. The management and treatment of DR have been a research hotspot and difficulty in recent years. In 2019, the American Academy of Ophthalmology issued the Diabetic Retinopathy Preferred Practice Pattern, describing the clinical treatment of DR [3]. The therapy methods currently available for DR include retinal laser photocoagulation, surgery, and drug therapy [4]. Nevertheless, laser therapy destroys partial retinal cells and is accompanied by vitreous hemorrhage [5]. Vitrectomy is likely to cause complications such as iatrogenic retinal rupture and cataracts [6]. For administration of anti-VEGF drugs, serious side effects including endophthalmitis, retinal detachment, and uveitis have been observed [7]. As a result, this study is aimed at exploring DR therapeutic candidates based on a systematic process.

The retinal capillary is the critical interface for nutrients, oxygen, and metabolites exchange between the nerve membrane and the circulation [8]. The gradual loss of retinal capillary perfusion causes retinal vascular degeneration [9]. Microvascular dysfunction is the main pathological feature of DR [10]. Human retinal microvascular endothelial cells (HRMECs) are tightly connected to the junction and located in the inner wall of the retinal capillary [11]. HRMECs secrete a series of vasoactive substances and cytokines to maintain vascular endothelial barrier function. Hyperperfusion and resultant shear stress of DR directly provoke HRMECs apoptosis and capillary occlusion [12]. Accordingly, HRMECs injury is the fundamental pathological basis of microvascular dysfunction [13] and the carrier for the systematic process of candidates discovery.

The factors causing HRMECs damage are complicated. Oxidative stress, cytokines, advanced glycosylation end products, polyol pathway, and protein kinase C are related to microvascular endothelial injury [14, 15]. Clinical research revealed that oxidative stress occurs in the eyes of DR patients, and the production of free radicals leads to the imbalance of the retinal antioxidant system [16, 17]. In addition, multiple factors can promote retinal oxidative stress. On the contrary, oxidative stress strengthens the abnormalities of multiple factors, thus forming a vicious cycle [18]. Therefore, inhibiting HRMECs oxidative stress injury is a promising strategy for the DR candidates discovery.

Molecular switches are critical targets for controlling a series of intracellular signal transduction reactions [19]. The recognition of molecular switches can interpret disease regulation and provide a practical and reliable basis for clinical treatment. As one of the initiating factors, oxidative stress has been confirmed to cause HRMECs injury through crosstalk [20]. However, the pathogenesis and molecular switches of oxidative stress-induced HRMECs remain unclear. The analysis for molecular switches is of great significance for elucidating the pathogenesis of HRMECs oxidative stress injury and screening effective ingredients. In brief, the identification of molecular switches is a prerequisite for screening DR therapeutic candidates.

In the present study, HRMECs were induced by tert-butyl hydroperoxide (t-BHP) to simulate the retinal microvascular oxidative stress injury in vitro [21]. Then, the molecular switches in the HRMECs oxidative stress injury model were identified by integrating functional genomics and network pharmacology techniques. Furthermore, combining high-throughput screening and pharmacodynamic activity evaluation experiments, a set of activity evaluation systems was built, and potential therapeutic candidates were obtained. Finally, the candidate was applied in the HRMECs oxidative stress model to evaluate its potential effect and mechanism. The effectiveness of potential therapeutic candidates was proved, and the systematic process's accuracy was demonstrated.

## 2. Materials and Methods

### 2.1. Reagents

Tertiary butylhydroquinone (TBHQ) and t-BHP were obtained from Macklin (Shanghai, China). Fetal bovine serum (FBS) and penicillin-streptomycin (PS) were provided by Gibco BRL (Grand Island, NY, USA). Dulbecco's modified eagle medium (DMEM) was purchased from Corning (Corning, USA), and 3-(4,5-dimethyl-2-thiazolyl)-2,5-diphenyl-2-H-tetrazolium bromide (MTT) was acquired from Sigma-Aldrich (Madrid, Spain). Lactate dehydrogenase (LDH), Annexin V-FITC, superoxide dismutase (SOD), catalase (CAT), and glutathione peroxidase (GPx) assay kits were products of the Beyotime (Jiangsu, China). 1,1-Diphenyl-2-picrylhydrazyl (DPPH) detection kit was obtained from Nanjing Jiancheng (Nanjing, China). Primescript™ RT reagent kit and SYBR® Premix Ex Taq™ II kit were provided by Takara (Dalian, China). Antibodies for heme oxygenase 1 (HO-1) and sequestosome-1 (SQSTM1) were products of Proteintech (Rosemont, IL, USA), and an antibody for vascular endothelial cadherin (VE-cadherin) was purchased from Abcam (Cambridge, UK). TRIzol reagent was purchased from Invitrogen (Carlsbad, CA, USA).

### 2.2. Cell Culture and Model Construction

HRMECs and human embryonic kidney 293 cells (HEK293) were cultured in DMEM supplemented with 10% FBS and 1% PS. HRMECs were seeded in 96-well plates at 8 × 10^3^ and maintained at 37°C and 5% CO_2_. The model of retinal microvascular oxidative stress injury was established by t-BHP. After 4 h of culture, the cell viability was evaluated by MTT assay.

### 2.3. Transcriptome Sequencing and Network Pharmacology Analysis

#### 2.3.1. Analysis of Gene Differential Expression and Target Sets

Total RNA of HRMECs was isolated and purified by TRIzol reagent. CapitalBio Technology Inc. (Beijing, China) completed the construction and sequencing of the library. The quality of the RNA was detected with Agilent 2100 BioAnalyzer (Agilent Technologies, Santa Clara, CA, USA). The RNA was purified into mRNA by beads containing oligo (dT) and applied to construct the library by the NEB Next Ultra RNA Library Prep Kit. After constructing the cDNA library, the cDNA was sequenced on Illumina NovaSeq sequencer (Illumina, San Diego, CA, United States). Subsequently, StringTie [22] was applied to calculate the fragments per kilobase of exon per million mapped reads (FRKM), and the DESeq2 R package was used to perform the differential expressed genes (DEGs). Only genes with FPKM > 5, ∣log2FC | ≥1, and *P* ≤ 0.05 were considered statistically significant. In order to investigate the global function of DEGs, the biological processes and functional modules were analyzed. In the current research, DAVID (https://david.ncifcrf.gov/) [23] was adopted to obtain biological processes. STRING (https://string-db.org/cgi/input.pl) [24] and ClusterONE [25] were used to construct the protein interaction network (PIN) and mining the functional modules. According to the results of GO and module analysis, we divided the target sets.

#### 2.3.2. Cellular Pathway Network Construction and Molecular Switch Recognition

The cellular pathway networks are crucial to interpret the molecular mechanism of HRMECs oxidative stress injury. The pathway information involved in DEGs was obtained from KOBAS [26]. Based on the connectivity of target sets, the cellular pathway networks were constructed in Cytoscape [27]. The topological information of targets, such as degree, closeness, and betweenness, was acquired with CytoHubba [28]. Afterwards, the *Z* score formula was applied to normalize the topological parameters of targets and distinguish the molecular switch.

### 2.4. Luciferase Reporter Activity Assay

HEK293-antioxidant response element (ARE) cell line previously established by our research group [29] was incubated with 275 traditional Chinese medicine (TCM) ingredients (10 *μ*M). Flex station 3 (Molecular Devices, San Francisco, CA, USA) was applied to detect the luciferase activity and discover the potential active ingredients.

### 2.5. Cell Viability Evaluation

HRMECs were treated with different concentrations of andrographolide for 24 h, followed by 300 *μ*M t-BHP for 4 h. Then, HRMECs were incubated with 100 *μ*L MTT (0.5 mg/mL) for 4 h and added 150 *μ*L DMSO. Flex station 3 measured the absorbance at 490 nm to evaluate the cell viability.

### 2.6. Transcriptome and Network Pharmacology Analysis of Andrographolide

In order to further systematically reveal the molecular mechanism of the candidate, transcriptomics and network pharmacology were introduced. Total RNA of HRMECs treated by t-BHP with or without andrographolide was isolated. Transcriptomics was adopted to obtain the DEGs between the andrographolide and model group, and network pharmacology was applied to reveal the mechanism of DEGs. The analysis method is the same as 2.3.

### 2.7. DPPH Radical Scavenging Effect

The exogenous antioxidant activity of andrographolide was measured colourimetrically by DPPH assay. 40 *μ*L andrographolide was mixed with 60 *μ*L DPPH working solution at various concentrations (5 *μ*M-20 *μ*M). The mixture was incubated at room temperature for 30 min. The DPPH radical scavenging rate was calculated based on the absorbance detected at 517 nm.

### 2.8. Detection of SOD, CAT, and GPx

Antioxidant markers: SOD, CAT, and GPx activity reflect the endogenous antioxidant capacity of andrographolide. The pretreated HRMECs were lysed in RIPA buffer supplemented with 1% PMSF, and protein concentration was measured with the BCA protein assay kit. Then, we determined the activities of SOD, CAT, and GPx according to the SOD, CAT, and GPx manufacturer's protocols.

### 2.9. Lactate Dehydrogenase Assay

The pretreated HRMECs supernatant was collected. Mix lactic acid, INT solution, and enzyme solution to prepare the LDH detection working solution. 60 *μ*L cell supernatant and 120 *μ*L LDH detection solution were incubated in the dark for 30 min. The absorbance was measured at 490 nm, and the LDH release rate was calculated.

### 2.10. Cell Apoptosis Assay

HRMECs were added with 195 *μ*L Annexin V-FITC binding solution, followed by 5 *μ*L Annexin V-FITC and 10 *μ*L PI staining solution. After incubation at room temperature for 15 min, the cell apoptosis was detected by flow cytometry (BD Sciences, San Jose, CA, USA) or fluorescence microscope (LEICA, Solms, Germany).

### 2.11. Immunofluorescence Staining

HRMECs pretreatment with andrographolide were fixed by 4% paraformaldehyde, permeabilized with 0.1% Triton X-100, and blocked with 5% BSA. For VE-cadherin staining, HRMECs were incubated with rabbit anti-VE cadherin antibody (1 ∶ 1000) overnight and IgG H&L (Alexa Flour® 488) antibody (1 ∶ 2000) for 1 h. For filamentous actin (F-actin) staining, HRMECs were incubated with 100 nM FITC-conjugated phalloidin for 30 min. After staining, the nuclei was stained with Hoechest 33258 for 20 min and observed under the fluorescence microscope.

### 2.12. Real-Time Quantitative Polymerase Chain Reaction

After extracting total RNA from HRMECs, reverse transcription was conducted according to the instruction of the PrimeScript RT kit. The SYBR Premix Ex Taq reagent was used to detect mRNA expression, and the 2^−△△Ct^ method was applied to perform mRNA fold changes. The primer sequences of objective genes were presented in Table 1.

### 2.13. Western Blot Analysis

The protein extraction and concentration determination from HRMECs were operated as previously described. The equal amount of protein loaded on 8%-15% SDS-PAGE gels and electrotransferred to PVDF membrane. The membrane was blocked with 5% skimmed milk for 2 h, followed by incubation with primary antibodies overnight and HRP-conjugated IgG secondary antibodies for 2 h. Subsequently, the protein bands were visualized with ECL reagents under the Bio-Rad ChemiDocTM MP Imaging System (Hercules, CA, USA).

### 2.14. Molecular Docking

The direct inhibitory effect of andrographolide on the Nrf2-Keap1 PPI was explored by molecular docking. First, we selected the crystal structure of the receptor (PDB ID: 4L7B) in the PDB database (https://www.rcsb.org/). Then, the molecular structure of the andrographolide was downloaded from PubChem (https://www.ncbi.nlm.nih.gov/pccompound). CDOCKER carried out the molecular docking in Discovery Studio 4.0.

### 2.15. Molecular Dynamic Simulation

Molecular dynamic (MD) simulation was adopted to determine the interaction between andrographolide and Nrf2-Keap1 PPI (PDB ID : 4L7B). Ligand and protein topologies were generated by the GlycoBioChem PRODRG2 server (http://davapc1.bioch.dundee.ac.uk/cgi-bin/prodrg) and GROMOS96 43a1 force field, respectively. The equilibration steps were carried out into the combined stages of NVT (500 ps) and NPT (1000 ps). Then, we performed a 20 ns MD simulation on the complex. The simulation trajectory was analyzed by multiple parameters, such as root mean square displacement (RMSD), radius of gyration (Rg), root mean square fluctuations (RMSF), and total energy.

### 2.16. Statistical Analysis

All data were expressed as mean ± standard deviation (SD). One-way ANOVA analyzed the significance of the differences. *P* < 0.05 was considered to be statistically significant.

## 3. Results

### 3.1. t-BHP Induces HRMECs Damage

**Figure 1:**
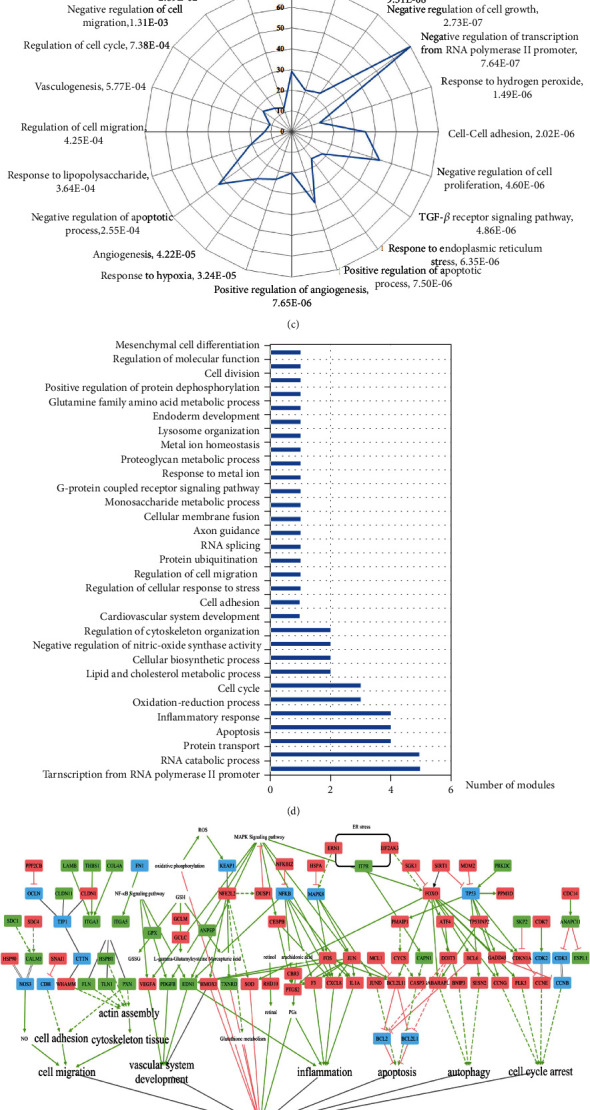
Effects of t-BHP on HRMECs. (a) t-BHP reduced HRMEC viability. Data are represented as mean ± SD (*n* = 3, ^###^*P* < 0.001 vs. control group). (b) The DEGs of t-BHP group vs. control group. Red nodes represent upregulated DEGs, and green nodes represent downregulated DEGs. (c) Gene Ontology enrichment results of DEGs regulated by t-BHP. The coordinate axis represents the number of DEGs. (d) Module analysis results of t-BHP on HRMECs. (e) Pathway network regulated by t-BHP. (f) Oxidative stress pathway network in t-BHP induced HRMECs. Rectangles: targets, red: upregulation, green: downregulation, blue: unknown-regulation; solid line: direct, dotted line: indirect, black solid line arrow: double regulation, black cross arrow: dissociation, and double black solid line: complex. Red stars as key targets.

As illustrated in Figure 1(a), t-BHP inhibited HRMECs growth in a dose-dependent manner. The HRMECs viability gradually decreased with the increase of t-BHP concentration. The cell viability decreased by nearly 50% of the maximum activity after treated with 300 *μ*mol·L^−1^ t-BHP for 4 h. It was used as the model induction condition for further study.

### 3.2. Molecular Switch of t-BHP-Induced HRMECs

The gene expression profiles of the control and the t-BHP group were constructed and compared. There were 980 DEGs between t-BHP and control group (FPKM > 5, ∣log2FC | ≥1, *P* ≤ 0.05), with 585 upregulated DEGs and 395 downregulated DEGs (Figure 1(b)). In order to obtain the overall function of DEGs, a total of 379 biological processes (Figure 1(c)) and 59 clusters (Figure 1(d)) were significantly annotated. Oxidative stress, vascular system development, cell proliferation, inflammation, cell migration, cell adhesion, cytoskeleton tissue, and nitric oxide (NO) regulation were pivotal in the HRMECs oxidative stress model. Then, we divided target sets and constructed the pathway network according to the key function (Figure 1(e)). We found that oxidative stress not only directly damaged HRMECs but also indirectly damaged HRMECs due to crosstalk. It suggested that oxidative stress is a pivotal factor in inducing HRMECs damage. As shown in Figure 1(f), nuclear factor-erythroid 2-related factor 2 (Nrf2) pathway, forkhead box O (FoxO) signaling pathway, GSH metabolism, mitochondrial oxidative phosphorylation, arachidonic acid metabolism, and retinol metabolism participated in HRMECs oxidative stress. To further focus on molecular switches, network topology analysis was used to identify critical targets. The results indicated that *NFE2L2* and *HMOX1* were the key genes, and the Nrf2 signaling pathway was the molecular switch in HRMECs oxidative stress (Figure 1(f)). The Keap1-Nrf2-ARE pathway could regulate the production of downstream antioxidant enzymes: *HMOX1*, thioredoxin reductase (*TXNRD*), *SOD*, *GPX*, glutamate-cysteine ligase regulatory (*GCLM*), and glutamate-cysteine ligase catalytic (*GCLC*) disrupt the antioxidant enzyme system, interfere with GSH metabolism, and thus, maintain the oxidative stress homeostasis of HRMECs.

### 3.3. Application of ARE Screening Model

**Figure 2:**
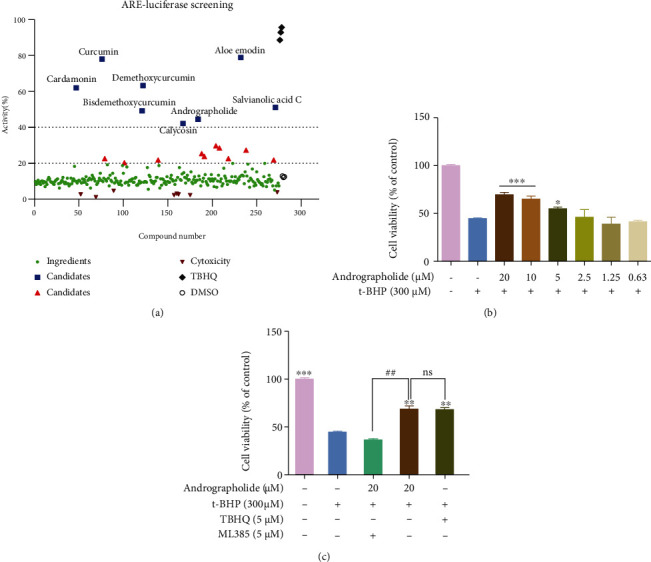
Screening results and validation of compounds. (a) Screening results of TCM compounds. (b) Protection of andrographolide on t-BHP-induced HRMEC injury model. (c) Effects of Nrf2 agonist and inhibitor on the HRMEC viability. Data are expressed as mean ± SD (*n* = 3, ^∗^*P* < 0.05, ^∗∗^*P* < 0.01, ^∗∗∗^*P* < 0.001 vs. t-BHP group; ^##^*P* < 0.01; ns: no significant difference vs. andrographolide group).

ARE contains the promoter region of a specific DNA sequence, which belongs downstream of Nrf2. Binding with ARE, Nrf2 maintains redox homeostasis by regulating the expression of downstream antioxidant enzymes [30, 31]. Hence, ARE was applied to identify the potential active ingredients of DR, and 275 TCM ingredients were screened based on the HEK293-ARE cell line (Figure 2(a)). In the luciferase reporter activity assay, 8 agonists exceeded 40% at 10 *μ*M concentration, and 18 agonists exceeded 20% at 10 *μ*M concentration. It noted that some agonists had been proved to play an essential role in DR. For instance, curcumin ameliorates diabetes-induced ultrastructure changes of the retina [32]. Aloe-emodin has an inhibitory effect on laser-induced choroidal neovascularization [33]. In addition, the research group has demonstrated the protective effect of carnosol on HMVEC oxidative stress damage [28]. These results suggested that the active ingredients have potential application prospects in the treatment of DR.

### 3.4. Andrographolide Improves Cell Viability in t-BHP Induced HRMECs

In order to demonstrate the effectiveness of ingredients and the accuracy of the systematic process, the candidate was applied in the HRMECs oxidative stress model. According to the principle of oral bioavailability and drug likeness, andrographolide was determined as the verification candidate. Andrographolide is a natural antioxidant, which is enormously significant for oxidative stress-induced diseases [34]. MTT assay showed that andrographolide (5 *μ*M-20 *μ*M) inhibited HRMECs oxidative stress injury (Figure 2(b)). Moreover, there was no significant difference in the protective effect between andrographolide and Nrf2 agonist TBHQ [35] (Figure 2(c)). The viability of HRMECs decreased after treated with andrographolide and Nrf2 inhibitor ML385 [36] (Figure 2(c)).

### 3.5. Transcriptomic Analysis Interprets the Critical Targets of Andrographolide on HRMECs

**Figure 3:**
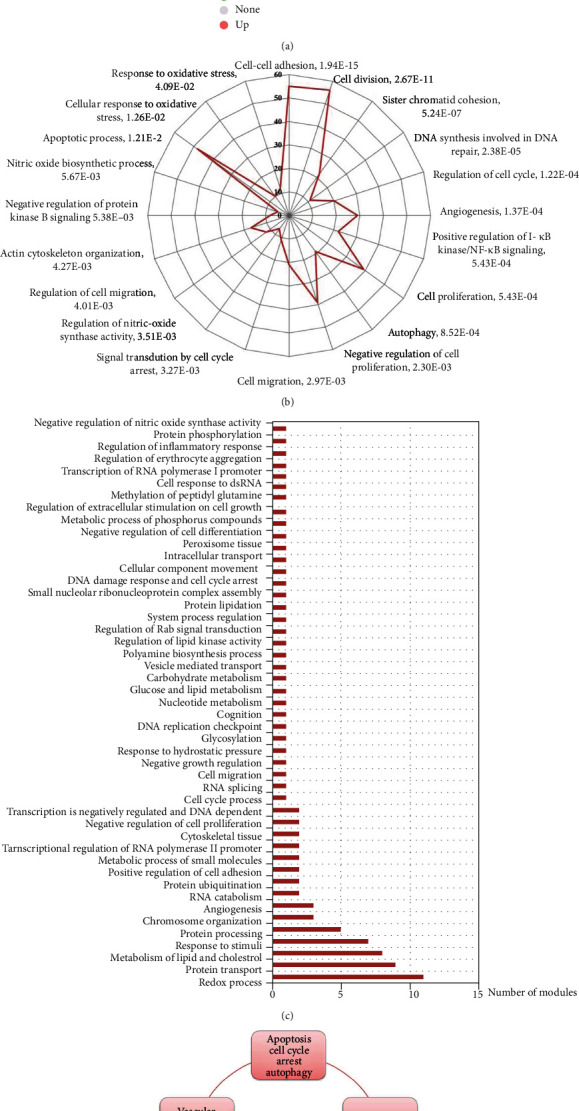
Transcriptomics analysis of the mechanism of andrographolide on HRMECs. (a) The DEGs of andrographolide group vs. t-BHP group. (b) GO enrichment analysis of DEGs. (c) Module identification of DEGs. (d) The target sets in andrographolide-mediated HRMECs.

**Figure 4:**
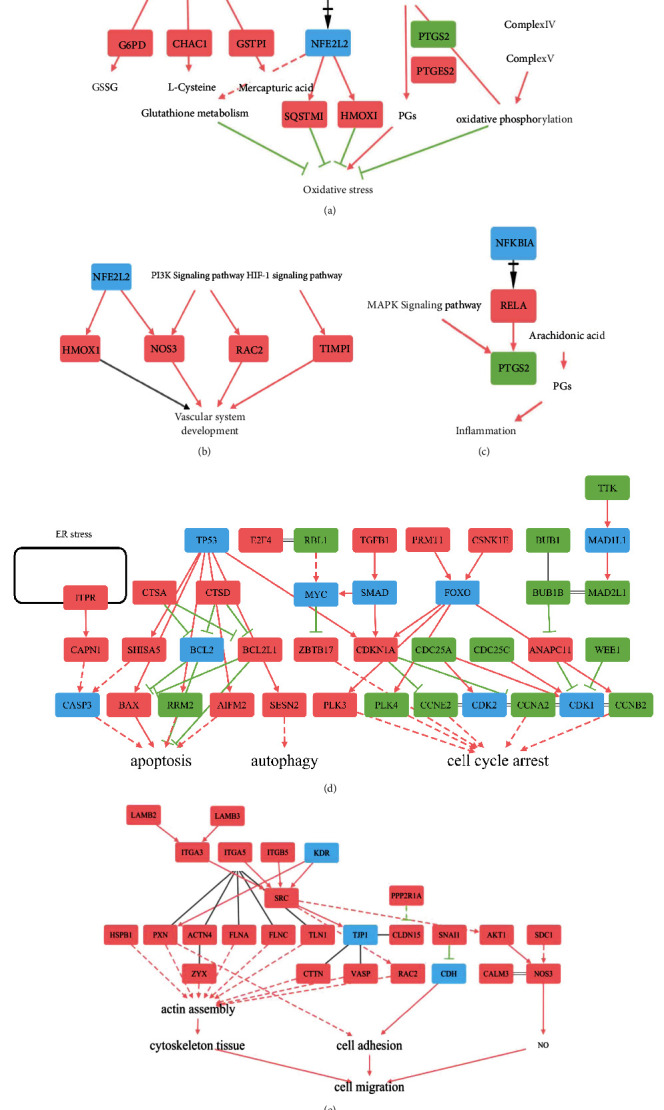
Pathway networks regulated by andrographolide. (a) The regulation of andrographolide on pathways in oxidative stress. (b) Vascular system development network regulated by andrographolide. (c) Regulation of andrographolide on inflammation pathway network. (d) The cell proliferation and death network regulated by andrographolide. (e) The regulation of andrographolide on pathways in cell migration. Rectangles: targets, red: upregulation, green: downregulation, blue: unknown regulation; solid line: direct, dotted line: indirect, black solid line arrow: double regulation, black cross arrow: dissociation, and double black solid line: complex.

Transcriptomics analysis was conducted to obtain the DEGs between andrographolide and model group. The volcano diagram showed that 1091 DEGs (940 upregulation and 151 downregulation) responded to andrographolide therapy (Figure 3(a)). Subsequently, GO enrichment and module analysis was further carried out. The GO enrichment (Figure 3(b)) and module analysis (Figure 3(c)) results revealed that the mechanism of andrographolide was associated with oxidative stress, vascular system development, apoptosis, cell cycle, autophagy, inflammation, and cell migration (Figure 3(d)). Furthermore, we divided the target sets and constructed the pathway networks. The oxidative stress pathway network showed that andrographolide modulated the Nrf2 signaling pathway, balanced glutathione metabolism, regulated arachidonic acid metabolism, and affected mitochondrial oxidative phosphorylation, thereby resisting HRMECs oxidative stress injury (Figure 4(a)). Andrographolide also affected vascular system development (Figure 4(b)) and inflammation (Figure 4(c)). Its mechanism was related to the regulation of *NOS3* and inflammatory factors. At the same time, andrographolide inhibited apoptosis, cell cycle, and autophagy (Figure 4(d)). In addition, andrographolide affected the expression of *NOS3*, *SNAI1*, and F-actin, thus promoting cell migration, regulating endothelial barrier, affecting cell adhesion, and inhibiting cytoskeleton damage (Figure 4(e)).

### 3.6. Andrographolide Inhibits HRMECs Oxidative Stress

**Figure 5:**
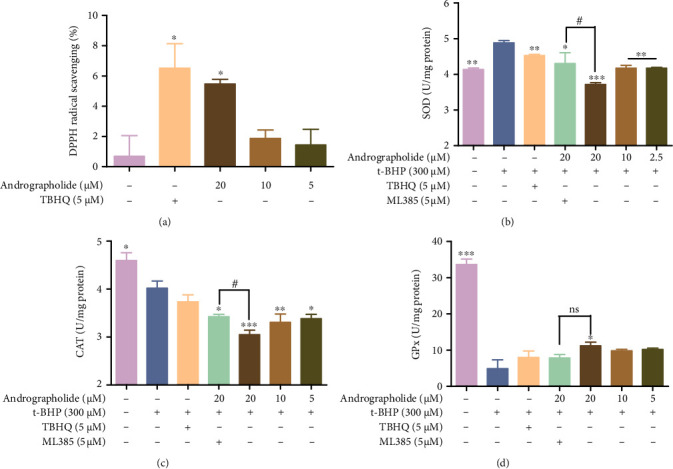
Antioxidant activity of andrographolide. (a) Scavenging effect of andrographolide on DPPH. Data are expressed as mean ± SD (*n* = 3, ^∗^*P* < 0.05 vs. control group). (b) Effect of andrographolide on SOD activity. (c) CAT activity regulated by andrographolide. (d) Regulation of andrographolide on GPx activity. Data are represented as mean ± SD (*n* = 3, ^∗^*P* < 0.05, ^∗∗^*P* < 0.01, ^∗∗∗^*P* < 0.001 vs. t-BHP group; ^#^*P* < 0.05; ns: no significant difference vs. andrographolide group).

**Figure 6:**
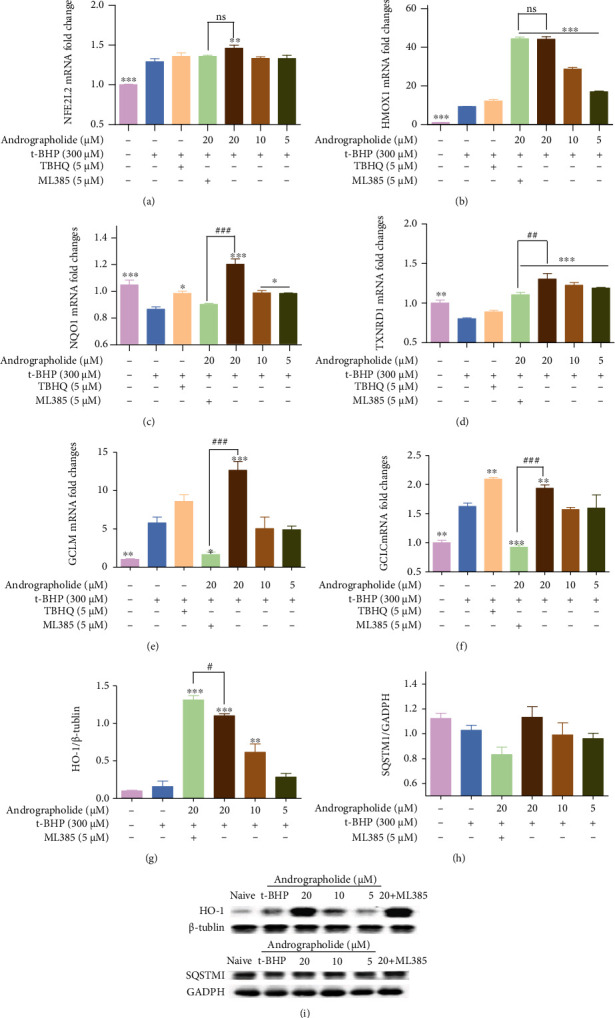
Effects of andrographolide on cytoprotective gene and protein expression in HRMECs. (a) *NFE2L2* mRNA, (b) *HMOX1* mRNA, (c) *NQO1* mRNA, (d) *TXNRD1* mRNA, (e) *GCLM* mRNA, and (f) *GCLC* mRNA. The mRNA levels were detected with RT-qPCR. *GAPDH* as an internal control. (g) HO-1 protein and (h) SQSTM1 protein. (i) The protein levels were detected with immunoblotting. The results were shown as mean ± SD (*n* = 3, ^∗^*P* < 0.05, ^∗∗^*P* < 0.01, ^∗∗∗^*P* < 0.001 vs. t-BHP group; ^##^*P* < 0.01, ^###^*P* < 0.001; ns: no significant difference vs. andrographolide group).

The endogenous and exogenous antioxidant capacity of andrographolide was evaluated. Andrographolide effectively scavenged DPPH free radicals (Figure 5(a)). The SOD activity increased, while the CAT and GPx activities decreased in HRMECs exposed to t-BHP. After andrographolide treatment, SOD and CAT activities decreased, GPx activity increased (Figures 5(b)–5(d)). Afterwards, the effects of the compound on Nrf2 and downstream molecular levels were detected by RT-qPCR and Western blot. As shown in Figure 6, the mRNA levels of *NFE2L2*, *HMOX1*, NADP (H) quinone oxidoreductase 1 (*NQO1*), *GCLM*, *GCLC*, *TXNRD1*, and the protein levels of HO-1 and SQSTM1 increased in the andrographolide treatment group. However, the difference of the SQSTM1 did not reach statistical significance. ML385 reversed the effect of andrographolide on the oxidative stress markers.

### 3.7. Andrographolide Interrupts Nrf2-Keap1 PPI

**Figure 7:**
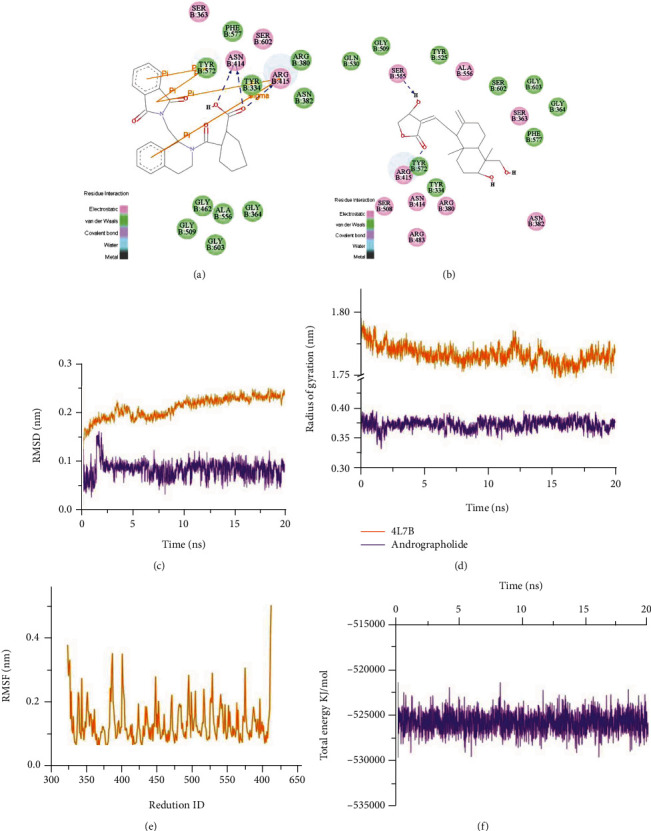
The results of molecular docking and molecular dynamics simulation. Molecular docking pattern diagrams of ligands with 4L7B. (a) Primary ligand and 4L7B. (b) Andrographolide and 4L7B. Analysis of MD trajectories generated by Gromacs. Trajectories for (c) RMSD, (d) Rg, (e) RMSF, and (f) total energy.

The interaction between andrographolide and Nrf2-Keap1 was evaluated by molecular docking. The results proved that andrographolide could directly inhibit the Nrf2-Keap1 PPI. Figures 7(a) and 7(b) intuitively shows the interaction between receptors and ligands, as well as specific amino acid binding sites. We found that andrographolide formed hydrogen bond interaction with residue ARG415 of 4L7B and performed hydrophobic interaction with ALA556 and TYR572 of 4L7B. In particular, the interaction pattern was similar to the primary ligand, indicating that andrographolide has a high potential inhibitory activity on Nrf2-Keap1. The optimal conformation of andrographolide and Nrf2-Keap1 obtained from docking results was used to MD. The stability of the system was further assessed using RMSD and Rg. Figures 7(c) and 7(d) illustrated that the complex reached equilibrium after 9 ns, representing the stability of the system. RMSF was further calculated to evaluate the flexibility of the residues. According to the analysis of fluctuation score at residues (Figure 7(e)), residues exhibited the low fluctuation in the system. In addition, the total energy was stably maintained in the systems, which approximated −525000 KJ/mol (Figure 7(f)).

### 3.8. Andrographolide Protects the Retinal Microvascular System and Suppresses t-BHP Induced Apoptosis

**Figure 8:**
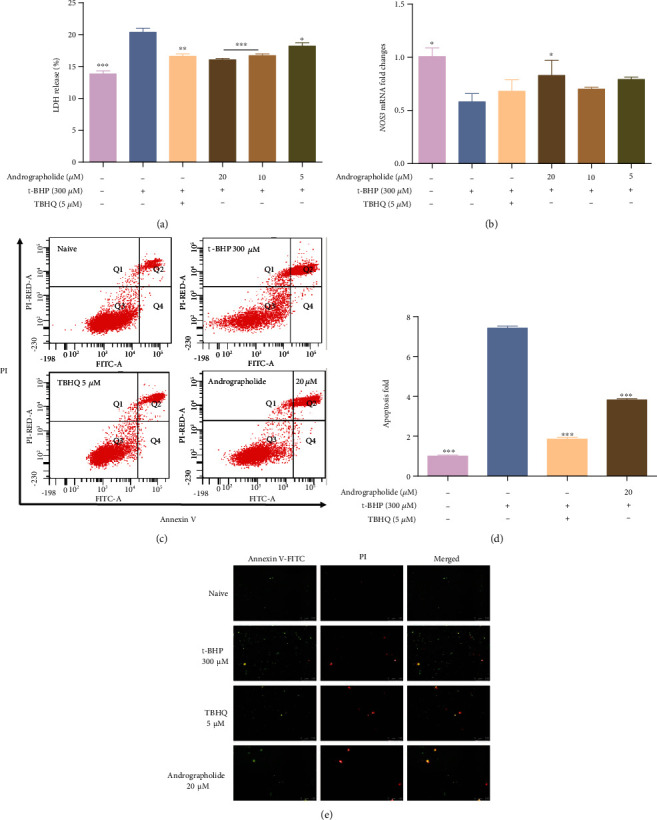
The influence of andrographolide on the retinal microvascular system. (a) The levels of the LDH release were measured using LDH kits. (b) The *NOS3* mRNA levels of cytoprotective genes. The value represents the means ± SD (*n* = 3, ^∗^*P* < 0.05, ^∗∗^*P* < 0.01, ^∗∗∗^*P* < 0.001 vs. t-BHP group). (c) Flow cytometry of apoptosis. Q2 and Q4 regions were apoptotic cells. (d) Detection of apoptosis by flow cytometry. Data are expressed as mean ± SD (*n* = 3, ^∗∗∗^*P* < 0.001 vs. t-BHP group). (e) Double staining of apoptosis (×200). Apoptotic cells were stained with green fluorescence, necrotic cells were stained with green and red fluorescence, and normal cells were not stained with fluorescence.

LDH reflects the membrane damage of HRMECs [37], endothelial nitric oxide synthase (eNOS) is crucial in vascular leakage and endothelial junction stability [38]. LDH release upregulated, and *NOS3* mRNA expression downregulated by t-BHP. Moreover, LDH release decreased, and *NOS3* mRNA expression increased by andrographolide (Figures 8(a) and 8(b)). The apoptosis of HRMECs was observed and analyzed by fluorescence microscope and flow cytometry. As shown in Figures 8(c)–8(e), t-BHP increased HRMECs apoptosis and necrosis. Andrographolide significantly inhibited HRMECs apoptosis.

### 3.9. Effects of Andrographolide on Inflammation, Cell Adhesion, and Cytoskeleton

**Figure 9:**
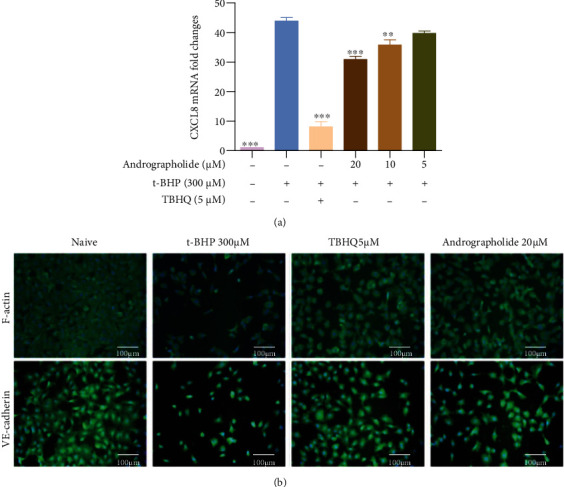
Andrographolide inhibited inflammatory response and regulated cell adhesion and cytoskeleton in t-BHP induced HRMECs. (a) Andrographolide significantly affected the *CXCL8* mRNA levels. Data are expressed as mean ± SD (*n* = 3, ^∗∗^*P* < 0.01, ^∗∗∗^*P* < 0.001 vs. t-BHP group). (b) Immunofluorescence staining of F-actin and VE-cadherin (×200). The nucleus showed blue fluorescence, and the F-actin or VE-cadherin showed green fluorescence.

The level of proinflammatory cytokines IL-8 is closely related to microvascular disease. As shown in Figure 9(a), after stimulation with t-BHP, the expression of *CXCL8* mRNA significantly upregulated. Andrographolide abolished the effect of *CXCL8*. Andrographolide on F-actin and VE-cadherin localization results showed in Figure 9(b). In the control group, F-actin was mainly surrounded by cells and arranged tightly without the apparent gap. After t-BHP induction, F-actin shortened to the cell center, with irregular aggregation and ectopic distribution. Similarly, t-BHP disrupted the distribution and expression of VE-cadherin on the cell membrane. In contrast, andrographolide significantly promoted the expression of VE-cadherin and affected the distribution of F-actin.

## 4. Discussion

DR causes irreversible damage to visual function and brings a heavy burden to society [39]. Preventing DR has become the top priority in the treatment of blindness. In the present study, we applied a systematic process to discover DR candidates.

Retinal microvascular dysfunction is one of the prominent metabolic abnormalities in DR. Inhibiting the HRMECs oxidative stress is an effective strategy for DR therapy. T-BHP is a free radical initiator and belongs to ROS. In this research, t-BHP damaged intracellular macromolecules and resulted in HRMECs death. Cell viability results proved that the HRMECs model could imitate the retinal microvascular injury environment to a certain extent.

Then, based on the HRMECs oxidative stress injury model, the mechanism was identified by multidisciplinary techniques. We successfully found that oxidative stress directly damaged HRMECs and indirectly damaged HRMECs due to crosstalk. The crosstalk was associated with vascular system development, cell proliferation, inflammation, cell migration, cell adhesion, and cytoskeleton tissue. The molecular switch is conducive to identifying active ingredients. Subsequently, the molecular switch of HRMECs oxidative stress injury model was distinguished by topological analysis. We showed that *NFE2L2* and *HMOX1* were crucial DEGs, and the Nrf2 signaling pathway was the critical molecular switch. In the oxidative stress injury model of HRMECs, the Keap1-Nrf2-ARE signaling pathway was activated, interfering with GSH metabolic balance, indirectly modulating SIRT1/FoxO signaling pathway, and inducing downstream antioxidant enzymes. The systematic process explored the vital targets to recognize active TCM ingredients of DR.

ARE plays a vital role in the Nrf2 signaling pathway. ARE regulates antioxidant system homeostasis by inducing Nrf2 downstream antioxidant genes [40]. The ARE-mediated reporting structure is widely used, including exogenous analysis and Nrf2 activator screening. In our previous study, ARE and luciferase reporter genes were connected to establish the HEK293-ARE overexpression cell line [28]. In the HEK293-ARE cell line, 275 TCM ingredients were screened, and 18 active ingredients were obtained. In the meantime, it has been reported that partial ingredients have potential therapeutic effects on microvascular diseases, such as curcumin, aloe-emodin, and carnosol. The results preliminarily proved that 18 candidates had a potential therapeutic effect on DR.

**Figure 10:**
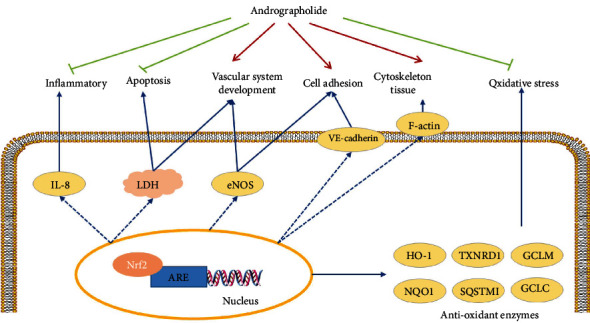
The mechanism of andrographolide inhibiting HRMEC injury. Red indicates promotion, and green indicates inhibition.

Andrographolide is a diterpene lactone [41], with multiple pharmacological effects such as antioxidant, anti-inflammatory, and antiviral [42]. In this study, andrographolide was found to increase the activity of ARE luciferase. Additionally, andrographolide has good oral bioavailability and high drug-likeness. In order to prove the potential therapeutic effect of 18 candidates for DR, the action of andrographolide on HRMECs was evaluated. Our data showed that andrographolide effectively inhibited t-BHP-induced oxidative stress damage of HRMECs. The transcriptomics and molecular biology results demonstrated that its mechanism was related to oxidative stress, vascular system development, apoptosis, cycle, autophagy, inflammation, migration, adhesion, and skeleton tissue (Figure 10). Inhibition the oxidative stress is one of the critical mechanisms of andrographolide. Andrographolide attenuated the HRMECs oxidative stress by inhibiting the production of DPPH free radicals and balancing CAT, SOD, and GPx. The Keap1-Nrf2-ARE pathway is essential for maintaining redox [43]. RT-qPCR and Western blot revealed that andrographolide inhibited HRMECs oxidative stress injury by promoting the expression of Keap1-Nrf2-ARE downstream antioxidant enzymes. However, the difference of the SQSTM1 did not reach statistical significance, which indicated that SQSTM1 might not be the critical target for andrographolide. Molecular docking and molecular dynamic simulation demonstrated that andrographolide was an Nrf2 agonist, inhibiting the Keap1-Nrf2 complex. And the ARG415, ALA556, and TYR572 were the crucial amino acid residues. Subsequently, the Nrf2 signaling pathway was blocked or activated by Nrf2 inhibitor ML385 and Nrf2 agonist TBHQ. The Nrf2 signaling pathway was reversed demonstrated as the main pathway for andrographolide against HRMECs oxidative stress injury.

In addition, apoptosis of HRMECs is a critical pathological basis of DR [44]. As an indicator of cell membrane damage, LDH releases outside the cell membrane when HRMECs apoptosis [45]. *NOS3* causes changes in vascular permeability by promoting the formation of NO [46]. Andrographolide could reduce the release of LDH, promote the expression of *NOS3*, and inhibit the apoptosis of HRMECs. Excessive inflammation leads to long-term inflammation and cell damage. Various proinflammatory factors, such as IL-8 and TNF-*α*, have been identified at increased concentrations in the retina and vitreous [47]. Andrographolide could inhibit the expression of *CXCL8* mRNA and alleviate the inflammation of HRMECs. VE-cadherin is the downstream target of *SNAI1* and reflects microvascular endothelial function and vascular permeability [48]. F-actin is the main component of the cytoskeleton and participates in the maintenance of cell morphology and spatial structure [49]. Andrographolide regulated cytoskeleton organization and adhesion status by affecting the distribution of F-actin and the expression of VE-cadherin. Furthermore, recent studies have demonstrated that Nrf2 participates in the microvascular system, inflammatory, and cytoskeleton. Nrf2 activation inhibits endothelial cell apoptosis and inflammation [50–52]. The F-actin cytoskeleton is highly disorganized, which prevents Nrf2 binding [53]. As an Nrf2 agonist, andrographolide has regulatory effects on vascular system development, apoptosis, inflammation, adhesion, and skeletal tissue. We speculated that andrographolide cooperatively inhibited HRMECs injury by modulating the Nrf2 pathway.

In brief, we successfully found that andrographolide inhibited HRMECs injury through modulating the Nrf2 pathway. The mechanism of andrographolide is consistent with the molecular switch, which confirms the importance of the Nrf2 in HRMECs oxidative stress injury. Therefore, it is reasonable to speculate that 18 candidates have potential therapeutic effects on DR by modulating the Nrf2 pathway. However, further research is needed to translate experimental results into clinical results.

## 5. Conclusion

In this study, we discovered 18 DR candidates through a systematic process. Firstly, we revealed the molecular mechanism of HRMECs oxidative stress injury and identified Nrf2 as the molecular switch of HRMECs oxidative stress injury. Further, 18 potential Nrf2 agonists were obtained. Finally, the effect and mechanism of andrographolide against HRMECs oxidative stress injury were investigated. We initially explored that 18 candidates cooperatively inhibited HRMECs injury by modulating the Nrf2 pathway. Overall, this study discovered DR therapeutic candidates based on the molecular switch and provided an effective systematic process for other diseases.

## Figures and Tables

**Table 1:** Primers for RT-qPCR analyses.

Gene	Forward primer	Reverse primer
*HMOX1*	AAAGTGCAAGATTCTGCCCCC	CATGCCTGCATTCACATGGC
*NFE2L2*	TCTGCCAACTACTCCCAGGT	ACGTAGCCGAAGAAACCTCA
*NOS3*	GATGAGTATGACGTGGTGTCCC	CCGAGGGGAGCTGTTGTA
*GAPDH*	GGAGCGAGATCCCTCCAAAAT	GGCTGTTGTCATACTTCTCATGG
*NQO1*	AAAGGATGGGAGGTGGTGGAGTC	AATATCACAAGGTCTGCGGCTTCC
*TXNRD1*	CTGGAAGGAACGCTCTCGGAATTG	ACCTCCTGAGCCACCTCCAATG
*GCLM*	GCCTGTTCAGTCCTTGGAGTTGC	GTGCGCTTGAATGTCAGGAATGC
*GCLC*	GCACATCTACCACGCCGTCAAG	GCTGTCCTGGTGTCCCTTCAATC
*CXCL8*	CCTTCCTGATTTCTGCAGCTC	TTCTTGGATACCACAGAGAATGAA

## Data Availability

The data used to support the findings of this study are included within the article, and further details are available from the corresponding author upon request.

## Abbreviations

ARE: Antioxidant response element
CAT: Catalase
DEGs: Differential expressed genes
DMEM: Dulbecco's modified eagle medium
DPPH: 1,1-Diphenyl-2-picrylhydrazyl
DR: Diabetic retinopathy
eNOS: Endothelial nitric oxide synthase
F-actin: Filamentous actin
FBS: Fetal bovine serum
FoxO: Forkhead box O
FRKM: Fragments per kilobase of exon per million mapped reads
GCLC: Glutamate-cysteine ligase catalytic
GCLM: Glutamate-cysteine ligase regulatory
GPx: Glutathione peroxidase
HEK293: Human embryonic kidney cells
HO-1: Heme oxygenase 1
HRMECs: Human retinal microvascular endothelial cells
IL-8: Interleukin-8
LDH: Lactate dehydrogenase
MTT: 3-(4,5-dimethyl-2-thiazolyl)-2,5-diphenyl-2-H-tetrazolium bromide
NO: Nitric oxide
NQO1: NADP (H) quinone oxidoreductase 1
Nrf2: Nuclear factor-erythroid 2-related factor 2
PIN: Protein interaction network
PS: Penicillin-streptomycin
ROS: Reactive oxygen species
SOD: Superoxide dismutase
SQSTM1: Sequestosome-1
t-BHP: Tert-butyl hydroperoxide
TBHQ: Tertiary butylhydroquinone
TCM: Traditional Chinese medicine
TXNRD1: Thioredoxin reductase 1
VE-cadherin: Vascular endothelial cadherin.

## Supplementary Materials

Supplementary Materials The graphical abstract is shown in the supplementary figure 1. All transcriptome analysis data in the cell samples are shown in supplementary table 1 and supplementary table 2. There are 980 differential expressed genes between t-BHP and control group and 1091 differential expressed genes between t-BHP and andrographolide group.
