# Supplementary material for: Discovery of Therapeutic Candidates for Diabetic Retinopathy Based on Molecular Switch Analysis: Application of a Systematic Process
Source: Oxid Med Cell Longev. 2022 Jan 6;2022:3412032. doi: 10.1155/2022/3412032 (PMC8758313; doi:10.1155/2022/3412032)
Supplement: Supplementary Materials — The graphical abstract is shown in the supplementary figure 1. All transcriptome analysis data in the cell samples are shown in supplementary table 1 and supplementary table 2. There are 980 differential expressed genes between t-BHP and control group and 1091 differential expressed genes between t-BHP and andrographolide group. [file 3412032.f1.zip › supplementary figure 1.pptx]

## Slide 1
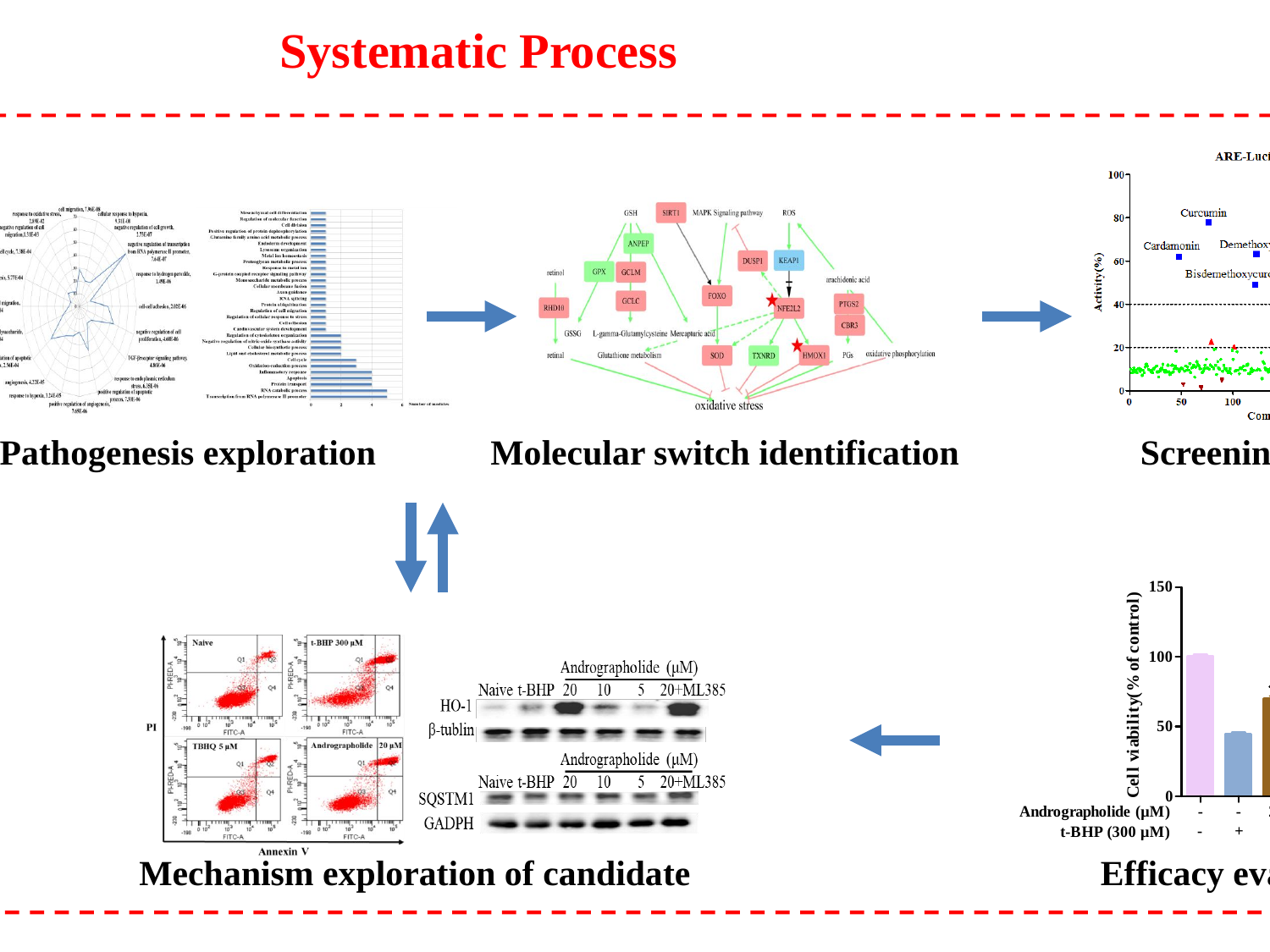

Systematic Process
Model construction
Pathogenesis exploration
Molecular switch identification
Screening of candidates
Mechanism exploration of candidate
Efficacy evaluation of candidate
